# An Electronic Health Record–Integrated Application for Standardizing Care and Monitoring Patients With Autosomal Dominant Polycystic Kidney Disease Enrolled in a Tolvaptan Clinic: Design and Implementation Study

**DOI:** 10.2196/50164

**Published:** 2024-05-01

**Authors:** Maroun Chedid, Fouad T Chebib, Erin Dahlen, Theodore Mueller, Theresa Schnell, Melissa Gay, Musab Hommos, Sundararaman Swaminathan, Arvind Garg, Michael Mao, Brigid Amberg, Kirk Balderes, Karen F Johnson, Alyssa Bishop, Jackqueline Kay Vaughn, Marie Hogan, Vicente Torres, Rajeev Chaudhry, Ziad Zoghby

**Affiliations:** 1 Hennepin Healthcare, Minneapolis, MN, United States; 2 Division of Nephrology and Hypertension, Department of Medicine, Mayo Clinic, Jacksonville, FL, United States; 3 Division of Nephrology and Hypertension, Department of Medicine, Mayo Clinic, Rochester, MN, United States; 4 Division of Nephrology and Hypertension, Department of Medicine, Mayo Clinic, Scottsdale, AZ, United States; 5 Division of Nephrology and Hypertension, Department of Medicine, Mayo Clinic, LaCrosse, WI, United States; 6 Division of Information Technology, Mayo Clinic, Rochester, MN, United States; 7 Division of Community Internal Medicine, Department of Medicine, Mayo Clinic, Rochester, MN, United States

**Keywords:** ADPKD, autosomal dominant polycystic kidney disease, polycystic kidney disease, tolvaptan, EHR, electronic health record, digital health solutions, monitoring, kidney disease, drug-related toxicity, digital application, management, chronic disease

## Abstract

**Background:**

Tolvaptan is the only US Food and Drug Administration–approved drug to slow the progression of autosomal dominant polycystic kidney disease (ADPKD), but it requires strict clinical monitoring due to potential serious adverse events.

**Objective:**

We aimed to share our experience in developing and implementing an electronic health record (EHR)–based application to monitor patients with ADPKD who were initiated on tolvaptan.

**Methods:**

The application was developed in collaboration with clinical informatics professionals based on our clinical protocol with frequent laboratory test monitoring to detect early drug-related toxicity. The application streamlined the clinical workflow and enabled our nursing team to take appropriate actions in real time to prevent drug-related serious adverse events. We retrospectively analyzed the characteristics of the enrolled patients.

**Results:**

As of September 2022, a total of 214 patients were enrolled in the tolvaptan program across all Mayo Clinic sites. Of these, 126 were enrolled in the Tolvaptan Monitoring Registry application and 88 in the Past Tolvaptan Patients application. The mean age at enrollment was 43.1 (SD 9.9) years. A total of 20 (9.3%) patients developed liver toxicity, but only 5 (2.3%) had to discontinue the drug. The 2 EHR-based applications allowed consolidation of all necessary patient information and real-time data management at the individual or population level. This approach facilitated efficient staff workflow, monitoring of drug-related adverse events, and timely prescription renewal.

**Conclusions:**

Our study highlights the feasibility of integrating digital applications into the EHR workflow to facilitate efficient and safe care delivery for patients enrolled in a tolvaptan program. This workflow needs further validation but could be extended to other health care systems managing chronic diseases requiring drug monitoring.

## Introduction

Autosomal dominant polycystic kidney disease (ADPKD) is the leading genetic cause and the fourth overall cause of end-stage kidney failure (ESKF) [1]. Patients with polycystic kidney disease 1 (PKD1) mutations develop ESKF 20 years earlier than those with PKD2 mutations [2,3]. The Mayo imaging classification (MIC) is a validated tool that identifies patients at risk for rapid progression to kidney failure, and disease-modifying therapy is recommended for patients with class 1C, 1D, or 1E, who have higher total kidney volume (TKV) growth rates [4]. In 2018, tolvaptan (brand name Jynarque; Otsuka America Pharmaceutical) was approved by the US Food and Drug Administration (FDA) as the first drug to slow kidney function decline in patients with rapidly progressive ADPKD. Tolvaptan reduces kidney volume growth and estimated glomerular filtration rate (eGFR) decline, delaying the need for kidney replacement therapy [5,6]. Tolvaptan acts by blocking the vasopressin V2 receptors in the distal nephron and collecting duct, inhibiting urinary concentration and sodium reabsorption and reversing the tubuloglomerular feedback inhibition induced by vasopressin, thus acutely and reversibly decreasing eGFR and possibly glomerular hyperfiltration [5]. However, tolvaptan is associated with several side effects, including polyuria, urinary frequency, thirst, and nocturia, which require patient education on adequate hydration. Tolvaptan can also cause significant hepatotoxicity in 5% of patients; thus, periodic liver function tests are mandated by the FDA through the risk evaluation and mitigation strategy (REMS) safety program. Due to the side effects profile and the necessary frequent laboratory test monitoring, the cost associated with staff time to manage the program beyond face-to-face care can limit the ability of health care teams to safely provide this disease-modifying therapy [7,8].

Tools that are directly integrated in the electronic health record (EHR) workflow can increase efficiency, reduce cost, and improve drug monitoring and quality of care [9-12]. For example, a cluster randomized clinical trial in primary care provided access, within the EHR, to a prescription drug monitoring program (PDMP) before the prescription of opioids. The integration increased PDMP-querying rates, suggesting that direct access reduced hassle costs and could improve adherence to guideline-concordant care practices [13]. Another study reported that the design and implementation of an electronic registry with a complementary workflow established an active tracking system that improved monitoring of patients on anticoagulation therapy [14]. However, no prior EHR-integrated workflow has been developed and validated to safely and successfully monitor patients with ADPKD treated with tolvaptan.

This paper describes the design, development, and implementation of an intelligent automated application within the EHR to efficiently manage and monitor ADPKD patients enrolled in the Mayo Clinic tolvaptan program. The goal of this paper is to illustrate how digital applications integrated into the EHR workflow can facilitate efficient and safe care for patients enrolled in a drug monitoring program and how this workflow can be extended to similar programs in chronic disease management and lay the groundwork for quality improvement efforts.

## Methods

### Ethical Considerations

This work was reviewed by the Mayo Clinic Institutional Review Board (21-005428). The study was exempt from clinical research overight because it was considered to be a quality improvement project. Informed consent was waived and data were deidentified.

### Practice Setting

The Mayo Clinic is an integrated, multispecialty, multistate, large academic health system with locations in Minnesota, Florida, and Arizona; there are also other Mayo Clinic health system hospitals across Minnesota and Wisconsin. Since 2018, the Mayo Clinic uses a single, integrated EHR (Epic Systems) across all campuses. The Minnesota practice where the tolvaptan EHR application was initially launched includes 5 experienced nephrologists and 4 nurses directly involved in the ADPKD practice and various other specialists (ie, geneticists, hepatologists, liver surgeons, neurologists, neurosurgeons, pain specialists, interventional radiologists, transplant experts, and research coordinators) who care for these patients as needed. The tolvaptan EHR application was eventually adopted enterprise-wide in 2022, although the workflow may differ slightly by site based on the specificity and resources available in each practice. The 3 main Mayo Clinic campuses in Minnesota, Florida, and Arizona are designated as centers of excellence for ADPKD care by the Polycystic Kidney Disease Foundation.

### Clinical Protocol—Indications and Monitoring of Tolvaptan Treatment

Tolvaptan is prescribed in patients aged 18 to 55 years with eGFR ≥25 mL/min/1.73 m^2^ and at risk of rapid progression, defined by having an age-indexed height-adjusted TKV within MIC class 1C, 1D, and 1E [5,6,15]. Contraindications to initiate tolvaptan include history of liver injury, uncorrected hypernatremia, hypovolemia, inability to sense thirst, urinary tract obstruction, and concomitant use of strong CYP3A (cytochrome P450, family 3, subfamily A) enzyme inhibitors [16]. Tolvaptan initiation requires a multidisciplinary approach led by the treating nephrologist and a well-trained nursing team. In our program, following a shared decision discussion, eligible patients who agree to start tolvaptan are referred to a specialized nephrology nurse for a detailed educational session. The nurse visit includes a blood pressure check, assessment of alcohol consumption, dietary review, and in-depth education about the side effects of tolvaptan and the need for routine laboratory test monitoring. Patients are instructed to have a drug holiday in certain situations, such when they are unable to maintain adequate fluid intake, are hospitalized, are about to undergo an elective procedure, or are traveling. After confirming their willingness to take the medication, the nurse enrolls the patient in the mandatory REMS program, a drug safety program developed by the FDA for certain medications with serious safety concerns. As part of the tolvaptan REMS program, the following laboratory tests are performed before the morning dose of tolvaptan: aspartate transaminase (AST), alanine transaminase (ALT), total bilirubin, serum sodium (advised but optional), and creatinine (advised but optional). Results are collected 2 and 4 weeks after tolvaptan initiation, then monthly for 18 months, and every 3 months thereafter [16]. Staff must log in to complete a REMS attestation every 3 months for each patient. Liver enzyme elevation and changes in serum sodium or creatinine are reviewed after each test in a timely fashion by the nursing team and the prescribing nephrologist. This process is designed to detect any laboratory test abnormality or the development of drug complications that could otherwise go unnoticed. For example, one threshold for suspending the medication is elevation in AST or ALT twice above their baseline level, which might not be flagged in the test report. However, this process can be very cumbersome and time consuming for the clinical team. An intelligent, automated, and streamlined real-time EHR-based process of tracking and monitoring is essential for efficient and safe care delivery, especially in specialized centers with a large patient population.

### Architecture and Application Development by the Cohort Knowledge Intelligence Solutions Team

At the Mayo Clinic, the Cohort Knowledge Intelligence Solutions (CKIS) team is behind the development of many innovative patient cohort management solutions using the Epic Healthy Planet module. The CKIS team uses a collaborative, agile approach that incorporates feedback from clinical stakeholders and informatics to create care management solutions based on agreed-upon protocols of care that improve and automate processes for clinical staff, all managed within the EHR. All projects are reviewed through a standard intake process that factors in the scope of the project, enterprise impact, patient safety, quality of care, and revenue impact, among other criteria. Once approved and assigned, a business analyst and a builder will work with a group of stakeholders anywhere between several weeks to several months, depending on the scope of the project, to complete a solution build.

After the scope of a project is defined, a registry is used to gather a patient cohort along with a subset of metrics required to support the practice needs. The registry is an internal tool housed within Epic’s software and uses a rule-based framework consisting of 2 main components: an inclusion rule and metrics. The inclusion rule is used to define the population and uses a combination of charted data, such as the patient’s diagnosis, medication, and surgeries, or general demographics (eg, age and gender). The metrics (ie, rules) define what data will be captured for the population. Once a patient meets the defined inclusion criteria, the underlying metrics are processed, and data is captured. Metrics are designed to support the monitoring workflow in addition to future quality analysis and outcomes. They typically capture dates, laboratory test values, appointment information, patient demographics, and more. Finally, a report is built allowing users to visualize and interact with the registry data. Within the reports, specific patient metrics are displayed pertinent to the practice and may include laboratory test results, appointment information, or customized algorithms to create alerts for care team members to help them prioritize their work. The last phase of the build process includes testing and ensuring that the initial agreed-upon requirements have been met. Several months after the build is complete, the CKIS team meets with the customers to complete a value assessment that measures the impact of the solution provided.

### Process of Tolvaptan Application Development

The nephrology ADPKD practice assembled a team of stakeholders to streamline the enrollment and monitoring of patients in the tolvaptan program. After an initial discussion in early 2020, the clinical team determined the content of the application. The stakeholders met on average every 2 weeks over a 3-month period to develop, in an iterative fashion, the initial application and test the efficiency of the system over the subsequent 3 to 4 months. The team determined that 2 applications were required to serve the clinical need. The first and main application, titled Tolvaptan Monitoring Registry, manages all patients actively treated with tolvaptan by consolidating all relevant information in one screen. Patients who discontinue tolvaptan are removed from the first application and automatically added to the second application, titled Past Tolvaptan Patients. The second application allows the care team to maintain a log of all past participants and record the reason for drug discontinuation, such as adverse effects or requiring renal replacement therapy.

## Results

In September 2020, 2 EHR-based applications for monitoring patients taking tolvaptan were activated for clinical use. The tolvaptan clinic was established 2 years earlier when the FDA approved tolvaptan for the treatment of ADPKD. All patients enrolled in the program prior to September 2020 (n=32) were retrospectively added to the tolvaptan monitoring application.

### Clinical Workflow Using the Tolvaptan Application

**Figure 1.**
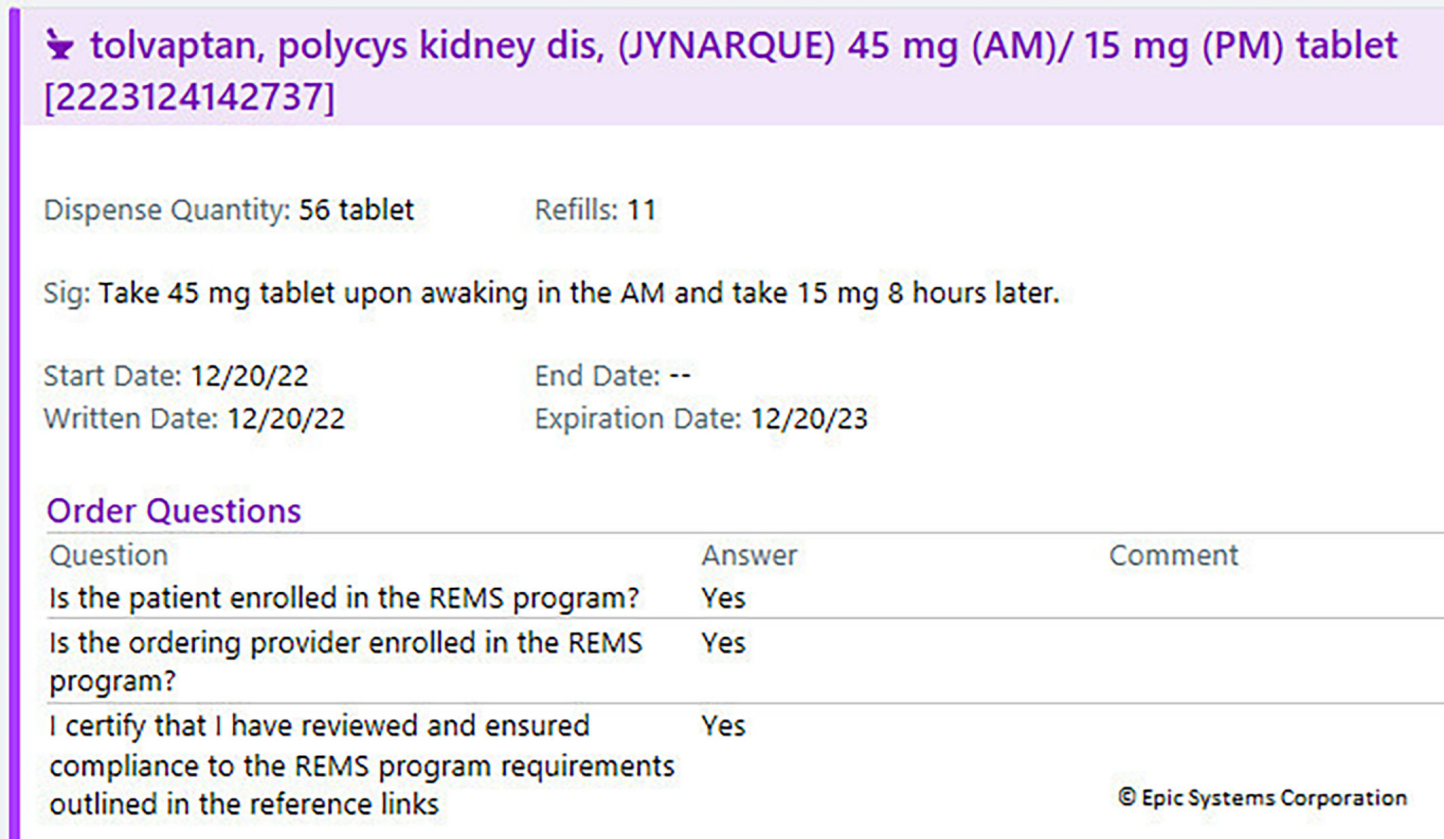
Tolvaptan order report. REMS: risk evaluation and mitigation strategy.

**Figure 2.**
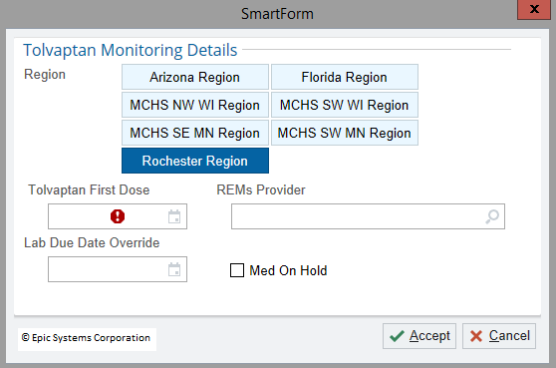
Smart form. MCHS: Mayo Clinic Health System; MN: Minnesota; NE: northeast; NW: northwest; REMS: risk evaluation and mitigation strategy; SW: southwest; WI: Wisconsin.

The tolvaptan application workflow involves the submission of an electronic prescription order by a nephrology nurse (Figure 1) and completion of an electronic activation form to enroll patients in the EHR-based tolvaptan monitoring application (Figure 2). This form includes the patient’s Mayo Clinic site, primary nephrology clinician, and date of treatment initiation. The automated addition of patients into the registry reduces the risk of missing any patient prescribed tolvaptan, ensuring that all treated patients are closely monitored for any adverse events that might occur while on therapy. Quarterly meetings take place between the nursing team and the nephrologists to review workflow issues and assess any new complications that may arise.

**Figure 3.**
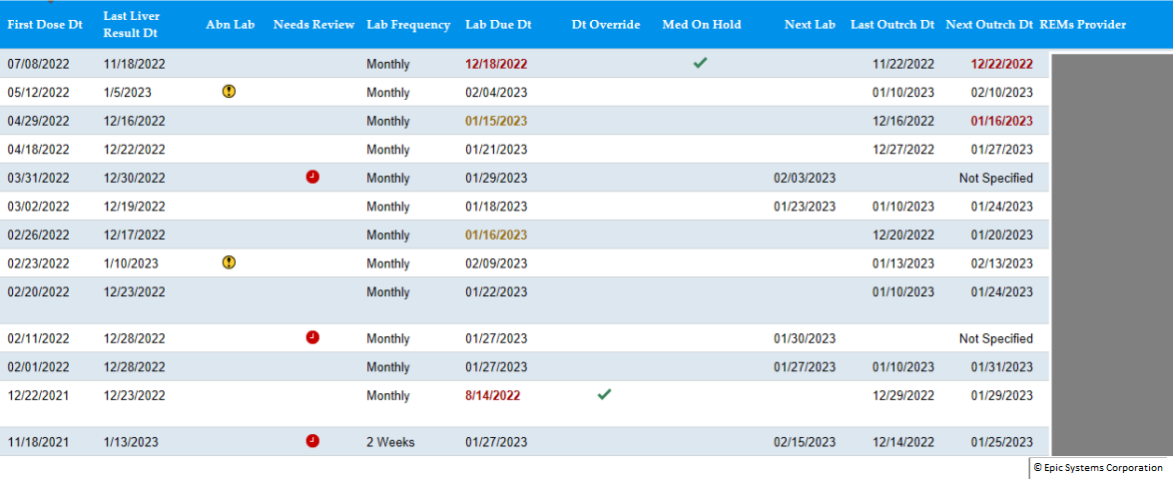
Tolvaptan monitoring snapshot. Abn: abnormal; Dt: date; REMS: risk evaluation and mitigation strategy.

**Figure 4.**
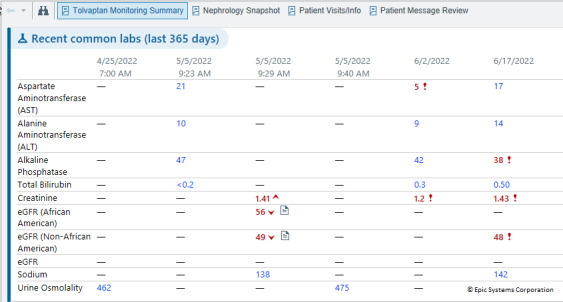
Tolvaptan Monitoring Summary. eGFR: estimated glomerular filtration rate.

**Figure 5.**
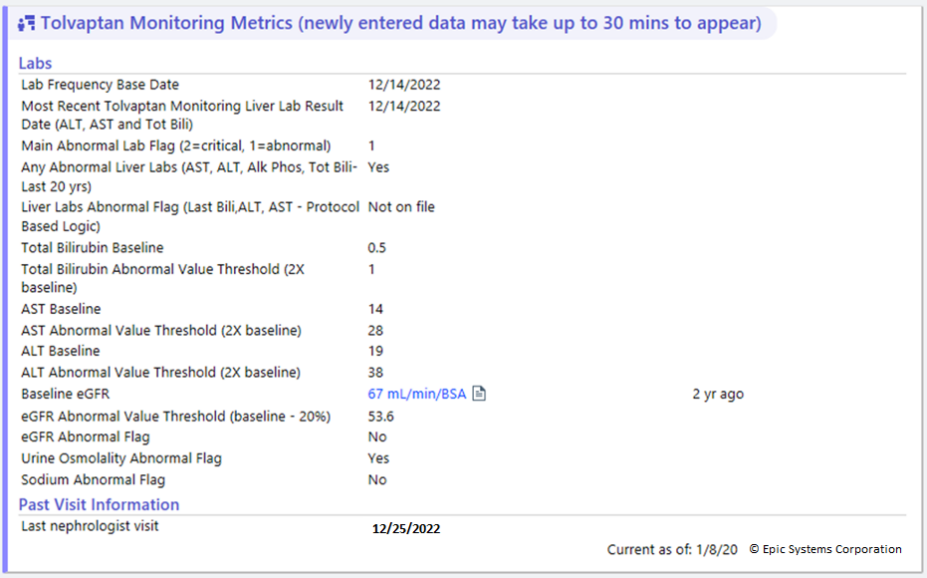
Tolvaptan Monitoring Metrics. ALK Phos: alkaline phosphatase; ALT: alanine transaminase; AST: aspartate aminotransferase; eGFR: estimated glomerular filtration rate; Tot Bili: total bilirubin.

**Figure 6.**
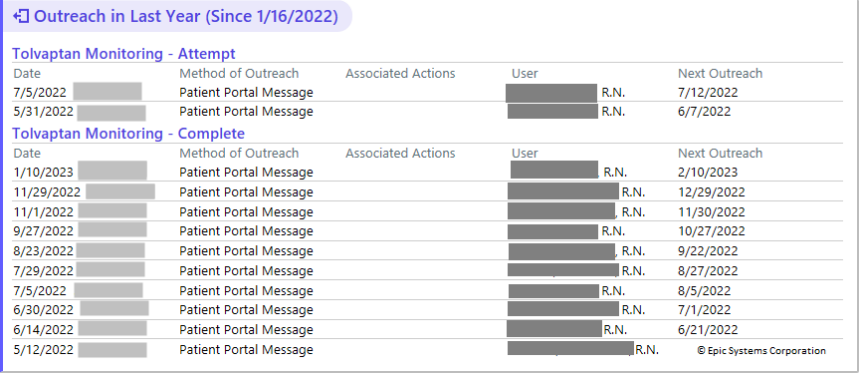
Tolvaptan detailed summary report.

The main application lists all patients actively taking tolvaptan and includes several columns with relevant information, such as the patient’s name, medical record number, date of initiation of tolvaptan, date of last liver function test, abnormal or urgent laboratory test flags, a “needs review” flag (Multimedia Appendix 1 provides details and flag criteria), the recommended laboratory test frequency based on the first dose date (ie, monthly or quarterly), the laboratory test’s due date and the date of the next scheduled laboratory test, the last and next (when applicable) nursing outreach dates to the patient, the name of the treating nephrologist, and the last clinic visit date (Figure 3). The application allows filtering based on these variables, such as visualizing only patients who have abnormal laboratory tests or need review based on new laboratory tests since the last outreach date. The application also provides more detailed information for a specific patient based on several reports embedded at the bottom of the screen. These include Tolvaptan Monitoring Summary, Patient Visits, Nephrology Notes/Orders, and Patient Message Review. In our clinical workflow, every week, 1 of 4 dedicated nurses (on a rotation basis) reviews all flagged patients and takes appropriate action based on our clinical protocol. The EHR-based tolvaptan monitoring application provides several reports that allow for a more detailed review of a specific patient without having to open their chart. The Tolvaptan Monitoring Summary displays all monitored laboratory test results, such as AST, ALT, bilirubin, serum creatinine, eGFR, serum sodium, and urine osmolarity (Figure 4). The Tolvaptan Monitoring Metrics window in the same section allows for quick access to recorded baseline laboratory test measurements and any abnormalities recorded. For example, the report displays a question and response: “Any Abnormal Liver Labs?” (answers are yes or no) (Figure 5). Additionally, all attempted or completed outreach interactions are listed with the name of the nurse conducting the activity and most recent nephrology note (Figure 6).

The Patient Visits report shows future scheduled appointments and surgeries, as well as a record of the patient’s last 10 outpatient visits. This report also includes the patient’s care team, demographics, and emergency contacts. The Nephrology Notes/Orders report displays pertinent medical history, current medication, immunizations, renal replacement therapy status, allergies, procedures, and the latest nephrology notes and specific ADPKD management–related comments by the nephrologist. Lastly, the Patient Message Review report includes all the patient’s communications with personnel, nurses, and clinicians, as well as patient online services.

### Characteristics of Enrolled Patients

As of September 1, 2022, a total of 214 patients have been enrolled in the tolvaptan program across the Mayo Clinic Health System in Minnesota, Arizona, Florida, and Wisconsin (Table 1).

Of the 214 patients, 126 (59%) were enrolled in the Tolvaptan Monitoring Registry, and the remaining 88 patients were included in Past Tolvaptan Patients. A total of 10 nephrologists were caring for these patients across the 4 locations. Table 2 displays characteristics of the patients in the Tolvaptan Monitoring Registry, including their demographics and MIC status.

The registry included 57.9% (n=124) female, 96.2% (n=206) White, 2.8% (n=6) Hispanic, and 0.9% (n=2) African American individuals. The mean age at enrollment was 43.1 (SD 9.9) years, and 86 patients had a documented MIC. Most patients had an MIC of 1C (n=38, 44.2%), followed by 1D (n=25, 29.1%) and 1E (n=19, 22.1%). Additionally, 3 patients (5.1%) had an MIC of 1B but were prescribed tolvaptan based on a non-MIC criterion. Of note, 33 (15.4%) patients were taking tolvaptan as part of a clinical trial and remained on the drug after trial completion (following FDA approval of the drug). These patients were added retrospectively to the application because they were taking tolvaptan prior to the creation of the EHR-based application.

**Table 1.** Distribution of patients with autosomal dominant polycystic kidney disease receiving tolvaptan treatment across the Mayo Clinic system.

Region	Tolvaptan Monitoring Registry (n=126), n (%)	Past Tolvaptan Patients (n=88), n (%)	Total (N=214), n (%)
Minnesota	80 (63.5)	75 (85.2)	155 (72.4)
Arizona	17 (13.5)	7 (8)	24 (11.2)
Florida	22 (17.4)	4 (4.5)	26 (12.1)
MCHS^a^, WI^b^	7 (5.6)	2 (2.3)	9 (4.2)

a MCHS: Mayo Clinic Health System.

b WI: Wisconsin.

**Table 2.** Patient demographics and Mayo imaging class.

			Tolvaptan Monitoring Registry	Past Tolvaptan Patients	Total patients
**Demographics**					
	Patients, n		126	88	214
	Female, n (%)		72 (57.1)	52 (59)	124 (58)
	Age at registry inclusion (years), mean (SD)		43.8 (9.9)	40.4 (9.9)	43.1 (9.9)
	White, n (%)		123 (97.6)	83 (94.3)	206 (96.3)
	Hispanic, n (%)		3 (2.4)	3 (3.4)	6 (2.8)
	African American, n (%)		0 (0)	2 (2.3)	2 (0.9)
	Enrollment through clinical trials, n (%)		29 (22.4)	4 (4.5)	33 (15.4)
**Mayo imaging class**					
	Patients, n		58	28	86
	1A		0 (0)	0 (0)	0 (0)
	1B		3 (5.2)	1 (3.6)	4 (4.7)
	1C		27 (46.6)	11 (39.3)	38 (44.2)
	1D		19 (32.8)	6 (21.4)	25 (29.1)
	1E		9 (15.5)	10 (35.7)	19 (22.1)

### Outcomes of Using the Tolvaptan Application

The implementation of the tolvaptan EHR-based application streamlined the monitoring process of patients treated with tolvaptan in several ways. First, the automated addition of patients into the registry reduced the risk of missing any patients started on tolvaptan, thus assuring that all treated patients were closely monitored for any adverse events that might occur while on therapy. Second, the application allowed for efficient and timely identification of patients who had abnormal laboratory test results and enabled nursing outreach to patients who might need further intervention or education on medication management. Overall, 20 (9.3%) patients had liver function test abnormalities, but only 5 (2.3%) had to discontinue the drug because of hepatotoxicity. The most common reason for drug discontinuation was related to the aquaretic effect, in 10 patients (4.7%), while only 4 (1.8%) could not continue in the program because of medical insurance–related issue. Third, the application provides a comprehensive and up-to-date summary of all pertinent clinical information related to the management of ADPKD, including medications, appointments, laboratory tests, and notes from the care team. Fourth, the application facilitated communication and collaboration among the multidisciplinary team involved in the care of patients with ADPKD. The standardization process and easy data access to all enrolled patients in the registry provided an opportunity for the care team to meet quarterly to review and discuss specific scenarios. These discussions sometimes led team members to share their experiences regarding challenging situations or drug-related adverse events or drug intolerance and at other times to propose enhancements to the EHR application. Finally, the application enhanced the efficiency of the tolvaptan program by reducing the time and effort (informally reported by the care team) required for enrollment, tracking, and monitoring of patients. More specifically, for the physicians, the only required task to enroll a patient in the program was identifying the candidate and making an electronic referral to the nursing team. The nursing team then initiated the education, treatment, and monitoring without any further escalation to the physician, unless there were concerns. For the nurses, all relevant information was consolidated, reducing the need to navigate to various EHR screens and modules.

## Discussion

### Principal Findings

In this report, we share our experience in developing and implementing an EHR application to manage and monitor patients with ADPKD who were enrolled in the tolvaptan program across several sites at our institution. This application streamlined the clinical workflow and enabled the nephrology nursing team to proactively take appropriate action to mitigate drug-related serious adverse events. Tolvaptan is the first and only FDA-approved drug to slow the progression of ADPKD, but it has multiple adverse effects, most seriously liver toxicity, which can be potentially severe, albeit rare. Therefore, frequent laboratory test monitoring is required to detect early drug-related toxicity. This application is crucial in facilitating the monitoring of patients taking tolvaptan, especially in large centers with high case load or smaller clinics with limited staff and resources. Overall, 20 (9.3%) patients had liver function test abnormalities, but only 5 (2.3%) had to discontinue the drug because of hepatotoxicity. The frequency of these events is very similar to those reported in the REPRISE clinical trial (10.9% hepatotoxicity and 1.6% discontinuation for a liver event) [6]. The most common reason for drug discontinuation was related to the aquaretic effect, which occurred in 4.7% (n=10) of patients. This is higher than the frequency reported in the REPRISE trial (2.1%) but not surprising since participants enrolled in clinical trials may be more motivated to adhere to the treatment protocol. Nonetheless, these clinical outcomes are reassuring.

The logistical requirements of any tolvaptan program may limit the ability of nephrology practices to provide this effective therapy. With the shortage of physicians and their high level of burnout [17-19], well-designed EHR integration that helps review, in a consolidated manner, relevant data for clinical care is important, although it comes with a higher up-front cost [20-23]. This is now more relevant because about 90% of office-based physicians in the United States use an EHR [24], and higher perceived EHR usability is associated with higher levels of perceived positive outcomes (improved patient care) and lower levels of perceived negative outcomes (worse patient interactions and work-life integration) [25]. Whether developing such digital systems is worth the investment is a relevant question for health care systems [26], but they can certainly be scaled in real-world settings. The cost of creating similar EHR-based applications will vary depending on each organization structure and is mostly an up-front cost. This includes the time required by both the clinical care team (nurses, physicians, and other clinical staff) and informatics team (program manager and technical build team) to identify the clinical need and develop and test the product. For our practice, it required at least 1 physician and 1 nurse champion to be present at each meeting (4 staff members were engaged) with the informatics team for 1 hour every 2 weeks on average over a 6-month period (12 hours per staff member involved). Since all our staff are salary based, this work was primarily supported by discretionary efforts and during nonclinical activities (lunch hour or administrative time). Regarding the informatics team (CKIS), our institution has allocated an operational budget to support various EHR-related projects across the enterprise; thus, we did not have to request extra funds to support this effort.

The advantages of these applications and data analytics capabilities within the EHR have been well described for various diseases and conditions, recently including more COVID-19–related activities, to manage the clinical practice safely [27-37]. Besides keeping track of a defined patient population, aggregating data, and identifying care gaps, communication with patients through the patient portal is readily accessible. In addition, bulk messaging (sending the same message to a group of patients in one click) is a convenient feature of the application. For example, staff can easily remind patients to do their monthly or quarterly laboratory tests when these have lapsed and do a synchronous or asynchronous quick health check if needed.

### Limitations

The design, development, and deployment in clinical practice of this integrated digital application has limitations. The process is iterative and requires buy-in from various stakeholders, an up-front investment in time, resources, and change management capabilities. Our clinical team was receptive, open to change, and willing to embrace new workflows because of the perceived value of adopting the application (more efficient and safer care delivery). One limitation of our study is that it was conducted in a single health care system. However, the successful implementation of this application in our Minnesota practice, followed by its expansion to all Mayo Clinic practices, highlights the potential for scaling to other health care systems. Another limitation of our study is the lack of objective efficiency outcome measures. The workflow improvement and satisfaction were not evalutated in a formal manner by the physicians and nurses. Ideally, our study would assess the impact of the application using (1) direct observation (time-motion studies), (2) EHR log-based analysis (EHR log data), (3) care team pre- and postimplementation surveys, or a combination of these. However, prior to the implementation of the application, the management of the tolvaptan program was ad hoc, carried out by a care team that performed multiple unrelated clinical activities. This made it impractical to use time-motion studies and impossible to meaningfully use EHR log data. A care team survey was considered, but because the transition to the application was done during the COVID-19 pandemic when our personnel resources were very strained, noncritical activities were paused. Prospective studies are necessary to validate the effectiveness of this application and its potential for improving care processes and ultimately patient outcomes.

### Conclusion

In conclusion, our multidisciplinary team developed an EHR-integrated digital monitoring protocol that could facilitate safe, efficient, and high-quality care for patients with ADPKD who were prescribed tolvaptan. The implementation of this application in our health care system can be scaled to other health care systems or smaller clinics after further validation. This can reduce some barriers and help safely provide the best available treatment for eligible patients.

## Supplementary material

10.2196/50164 Multimedia Appendix 1 Definitions of terms in columns and flags.

## Abbreviations

ADPKD: autosomal dominant polycystic kidney disease
ALT: alanine transaminase
AST: aspartate transaminase
CKIS: Cohort Knowledge Intelligent Solutions
CYP3A: cytochrome P450, family 3, subfamily A
eGFR: estimated glomerular filtration rate
EHR: electronic health record
ESKF: end-stage kidney failure
FDA: US Food and Drug Administration
MIC: Mayo imaging classification
PDMP: prescription drug monitoring program
PKD: polycystic kidney disease
REMS: risk evaluation and mitigation strategy
TKV: total kidney volume
