# Supplementary material for: An Electronic Health Record–Integrated Application for Standardizing Care and Monitoring Patients With Autosomal Dominant Polycystic Kidney Disease Enrolled in a Tolvaptan Clinic: Design and Implementation Study
Source: JMIR Med Inform. 2024 May 1;12:e50164. doi: 10.2196/50164 (PMC11085039; doi:10.2196/50164)
Supplement: Multimedia Appendix 1 [file medinform-v12-e50164-s001.docx]

**Appendix**

Definition of “Abnormal lab” column

This column displays a red (urgent) or yellow (abnormal) exclamation if the last laboratory tests within 14 days are abnormal according to the nephrology Tolvaptan practice protocols.

The following laboratory tests are evaluated: aspartate transaminase (AST), alanine transaminase (ALT), total bilirubin, alkaline phosphatase, serum sodium (Na), creatinine-based estimated glomerular filtration rate (eGFR) and urine osmolality. The rules are as follows:

Abnormal Flag definition
- Serum Na >/= 143 mmol/L or </= 134 mmol/L
- eGFR drops >/= 20% from baseline defined as the previous eGFR value
- Urine osmolality is > 280 mOsm/Kg (after the Tolvaptan start date)
- AST, ALT or total bilirubin are abnormal (based on Mayo Clinic laboratory reference values)

Urgent Flag definition
- AST, ALT, total bilirubin is >/= 3 times upper limit of normal (based on Mayo Clinic laboratory reference values)

Definition of “Needs Review column”

This column displays an icon when the most recent Tolvaptan laboratory test results date column is more recent than the last Outreach date column.
